# Expression of miR-93-5p as a Potential Predictor of the Severity of Chronic Thromboembolic Pulmonary Hypertension

**DOI:** 10.1155/2021/6634417

**Published:** 2021-04-17

**Authors:** Juanni Gong, Yuanhua Yang, Jianfeng Wang, Yidan Li, Xiaojuan Guo, Qiang Huang, Tuguang Kuang, Suqiao Yang, Jifeng Li, Ran Miao

**Affiliations:** ^1^Department of Respiratory and Critical Care Medicine, Beijing Chao-Yang Hospital, Capital Medical University, Beijing 100020, China; ^2^Key Laboratory of Respiratory and Pulmonary Circulation Disorders, Institute of Respiratory Medicine, Beijing 100020, China; ^3^Department of Interventional Radiology, Beijing Chao-Yang Hospital, Capital Medical University, Beijing 100020, China; ^4^Department of Echocardiography, Beijing Chao-Yang Hospital, Capital Medical University, Beijing 100020, China; ^5^Department of Radiology, Beijing Chao-Yang Hospital, Capital Medical University, Beijing 100020, China

## Abstract

**Background:**

MicroRNAs (miRNAs) play an important role in the pathogenesis of chronic thromboembolic pulmonary hypertension (CTEPH). However, the potential correlation between miRNA expression and the severity of CTEPH remains unclear. Our previous study indicated that miRNAs hsa-let-7b-3p, hsa-miR-17-5p, hsa-miR-106b-5p, hsa-miR-3202, hsa-miR-665, and hsa-miR-93-5p are closely involved in CTEPH. This study assessed the associations between the expression levels of these miRNAs and clinical parameters in CTEPH patients.

**Methods:**

A total of eight CTEPH patients and eight healthy adults as a reference group were included, and clinical data including total protein (TP), albumin (Alb), lactate dehydrogenase (LDH), hydroxybutyrate dehydrogenase (HBDH), uric acid (UA), and N-terminal pro-B-type natriuretic peptide (NT-proBNP) levels were collected. Right heart catheterization was conducted to obtain hemodynamic data including cardiac index (CI). The expression levels of let-7b-3p, miR-17-5p, miR-106b-5p, miR-3202, miR-665, and miR-93-5p were measured by quantitative real-time PCR (qPCR). Correlation analysis was applied to estimate the associations between miRNA expression levels and clinical parameters in CTEPH patients.

**Results:**

Serum TP and Alb levels were decreased, while LDH, HBDH, and UA levels were increased in CTEPH patients compared with the reference group (*P* < 0.05). miR-3202 and miR-665 were upregulated, whereas let-7b-3p, miR-17-5p, miR-106b-5p, and miR-93-5p were downregulated in CTEPH patients relative to the reference group (*P* < 0.05). miR-93-5p expression was positively correlated with NT-proBNP level and negatively correlated with CI (*P* < 0.05). Moreover, let-7b-3p tended to be positively correlated with mean pulmonary arterial pressure.

**Conclusions:**

miR-93-5p expression was associated with the severity of CTEPH and could act as a potential predictor of high-risk CTEPH.

## 1. Introduction

Chronic thromboembolic pulmonary hypertension (CTEPH), which manifests as progressive exertional dyspnea and right ventricular failure, is caused by pulmonary artery (PA) and pulmonary arteriole obstruction with proximal thromboembolism or distal vascular remodeling [1, 2]. The occurrence of CTEPH in pulmonary embolism survivors is approximately 3% [3]. However, severe CTEPH is associated with a high mortality rate of 24–67%, which increases over time [4]. A strategy for identifying high-risk CTEPH patients is therefore critical for improving the treatment and prognosis of these patients. However, predictive biomarkers for the severity and clinical outcomes of CTEPH are lacking.

Blood-derived biomarkers reflecting disease progression of CTEPH may provide potential predictive values. MicroRNAs (miRNAs) play critical roles in a variety of biological processes via the regulation of gene expression through mRNA inhibition or degradation at the posttranscriptional level [5]. Peripheral blood miRNAs are easy to collect and quantify, and they have become attractive circulating biomarkers for multiple diseases [6, 7]. For example, miRNAs have recently been identified as diagnostic and prognostic biomarkers for cardiovascular diseases including pulmonary hypertension (PH) [8]. miR-204 reflects PH severity, and miR-214-3p was confirmed to be a regulator of the hypoxia-induced proliferation and migration of pulmonary artery smooth muscle cells [9, 10]. Furthermore, miR-759 [11] and miR-22 [12] were reported as important indicators in the pathogenesis of CTEPH. In our previous study, we identified several critical miRNAs involved in the progression of CTEPH, including hsa-let-7b-3p, hsa-miR-17-5p, hsa-miR-106b-5p, hsa-miR-3202, hsa-miR-665, and hsa-miR-93-5p [13], indicating that these miRNAs might be useful in the diagnosis and treatment of CTEPH. Nevertheless, in-depth studies of the predictive values of these miRNAs remain insufficient. miR-223, miR-127, and miR-146a were shown to be positively associated with cardiovascular events independently of N-terminal pro-B-type natriuretic peptide (NT-proBNP) level and Framingham risk score even in asymptomatic individuals [14]. miR-424 (322) is upregulated in PH patients and linked with more serious symptoms and hemodynamics [15]. Taken together, research to date indicates that miRNAs may be correlated with clinical parameters reflecting disease severity in CTEPH patients and may serve as potential prognostic biomarkers.

The current study is aimed at detecting associations between the expression levels of miRNAs, including let-7b-3p, miR-17-5p, miR-106b-5p, miR-3202, miR-665, and miR-93-5p, and key clinical parameters in CTEPH patients. The predictive value of these miRNAs for the severity of CTEPH was determined.

## 2. Materials and Methods

### 2.1. Patient Recruitment

Eight CTEPH patients who were admitted at Beijing Chao-Yang Hospital from March to December 2017 and eight control subjects as a reference group were included. CTEPH was diagnosed based on the International Guidelines of Pulmonary Hypertension (2015 ERS/ESC guidelines for pulmonary hypertension) [16]. Briefly, CTEPH was defined by the following: (1) a mean pulmonary arterial pressure (mPAP) ≥ 25 mmHg and a pulmonary arterial wedge pressure (PAWP) ≤ 15 mmHg verified by right heart catheterization (RHC) and (2) chronic thrombosis with mismatched perfusion defects demonstrated by computed tomographic angiography or ventilation/perfusion scanning. In addition, the diagnostic criteria were met by right floating catheter, pulmonary ventilator-perfusion imaging, and/or spiral computed tomography pulmonary angiography. The exclusion criteria included hypertension, diabetes, coronary heart disease, cerebrovascular disease, malignancy, or other circulatory diseases, such as PH.

In the reference group, healthy adults who underwent routine physical examination were enrolled. The reference group had no history of heart, pulmonary, cancer, or thrombosis-related diseases. Additionally, no abnormality on laboratory measurements including routine blood tests, routine urine tests, biochemical tests, carcinoembryonic antigen and alpha fetoprotein levels, and chest radiographs was observed.

All patients were included on the date of diagnosis and had not been given PH-related treatment previously. Peripheral blood samples were obtained on the second day to ensure that CTEPH-related treatment had no effect on the expression of miRNAs.

### 2.2. Clinical Data Collection

Hemorheological and biochemical indexes reflecting the severity of CTEPH were collected. For CTEPH and control groups, blood parameters including hemoglobin (Hb) level, hematocrit (HCT) level, and platelet (Plt) count were tested. Additionally, biochemical indicators including total protein (TP) level, albumin (Alb) level, albumin : globulin (A : G) ratio, aspartate transaminase (AST) level, alanine transaminase (ALT) level, lactate dehydrogenase (LDH) level, hydroxybutyrate dehydrogenase (HBDH) level, total bilirubin (TBIL) level, direct bilirubin (DBIL) level, blood urea nitrogen (BUN) level, creatinine (Cr) level, and uric acid (UA) level were detected. For the CTEPH group, a number of specific clinical indicators were also collected, including World Health Organization function classification (WHO FC), medical history, 6-minute walk distance (6MWD), and NT-proBNP level.

### 2.3. RHC

RHC was performed for all CTEPH patients on the day of admission. The hemodynamic data were detected using a Philips monitoring system (Shenzhen Goldway Industrial Inc., China), and a Swan-Ganz catheter was used for the assessment of pressure in the right ventricle and pulmonary artery via the jugular vein. The mPAP and mixed venous oxygen saturation (SvO_2_) were recorded, and PAWP was calculated to exclude postcapillary pulmonary hypertension. Cardiac output (CO) was detected using thermodilution, and the average cardiac index (CI) of triplicate measurements was calculated. Pulmonary vascular resistance (PVR) was calculated using the following formula: PVR = (mPAP–PAWP)/CO.

### 2.4. Quantitative Analysis of miRNA Expression

Peripheral blood samples were collected from CTEPH patients and the reference subjects. Total RNA was extracted by RNA prep Pure Blood Kit (Tiangen Biotech Co., Ltd., China) following the manufacturer's protocol. Then, RNA was purified using the mirVana™ miRNA Isolation Kit (AM1561, Ambion, USA). The concentration and quality of RNA were detected by a Nanodrop ND1000 spectrophotometer (Thermo Fisher Scientific, USA) and an Agilent 2100 Bioanalyzer (Agilent Technologies, USA). The primer sequences for let-7b-3p, miR-106b-5p, miR-17-5p, miR-3202, miR-665, and miR-93-5p were designed using the Primer Premier 6.0 software (Premier Software Inc., USA) and produced by Sangon Biotech Co., Ltd (Shanghai, China) (Table S1). After conducting the reverse transcription reaction, quantitative real-time PCR (qPCR) analysis was performed using the SYBR Green master mix kit (Applied Biosystems, USA). The 20 *μ*L amplification reaction mixture contained the following: 1 *μ*L forward primer, 1 *μ*L reverse primer, 8 *μ*L cDNA template (at a constant concentration), and 10 *μ*L SYBR Premix Ex Taq (2×). The reaction program was as follows: 50°C for 3 min; 95°C for 3 min; 95°C for 10 s, 40 cycles at 60°C for 30 s; melt curve 60–95°C, with an increment of 0.5°C for 10 s plate read. U6 was used as the internal reference gene. All experiments were repeated three times to ensure accuracy.

### 2.5. Ethics Statement

This study was approved by the Ethics Committee of Beijing Chao-Yang Hospital, Capital Medical University. All procedures in studies involving human participants were performed in accordance with the ethics standards of the institutional and national research committee and with the 1964 Helsinki Declaration and its later amendments or comparable ethics standards. The need for written informed consent was waived, because the research involved no risk to the subjects and the waiver did not adversely affect the rights and welfare of the subjects.

### 2.6. Statistical Analysis

The results are presented as mean ± standard deviation for normally distributed data and as median and interquartile range for nonnormally distributed continuous variables. The data for categorical variables are expressed as percentages. Student's *t*-test was utilized for comparisons between the CTEPH and normal groups. To evaluate the potential value of miRNAs for estimating the severity of CTEPH, the associations between miRNA expression levels and clinical data were analyzed by Pearson correlation (continuous variables) or Spearman correlation (rank variables). Statistical significance was set at *P* < 0.05. Statistical analyses were performed with the SPSS 25.0 software (USA).

## 3. Results

### 3.1. Demographic and Clinical Data

A total of eight CTEPH patients (four males and four females) and eight healthy adults as a reference group (four males and four females) were recruited for this study. The median ages were 61.00 ± 6.82 years and 56.13 ± 4.49 years for the CTEPH patients and the reference group, respectively.

The CTEPH patients had significantly decreased serum TP (67.15 ± 4.56 vs. 76.79 ± 4.14 g/L, *P* < 0.05) and Alb (39.80 ± 2.88 vs. 46.09 ± 2.79 g/L, *P* < 0.05) levels and remarkably increased LDH (243.75 ± 45.96 vs. 180.16 ± 27.30 U/L, *P* < 0.05), HBDH (214.38 ± 40.20 vs. 153.00 ± 153.00 U/L, *P* < 0.05), and UA (447.87 ± 111.28 vs. 299.75 ± 76.62 U/L, *P* < 0.05) levels compared with the reference individuals. There were no significant differences in Hb, HCT, Plt, A : G, AST, ALT, TBIL, DBIL, BUN, and Cr levels between the two groups (all *P* > 0.05, Table 1).

Among the patients in the CTEPH group, five were classified as WHO FC I-II while three were classified as WHO FC III-IV. For this group, the mean disease duration was 43.88 ± 33.37 months. Also, the mean 6MWD was 390.06 ± 108.06 m, and the median NT-proBNP concentration was 1142.00 (145.42 and 2491.75) picograms per milliliter (Table 2).

### 3.2. Hemodynamic Data

All CTEPH patients underwent RHC, and the average values for mPAP, SvO_2_, CI, and PVR were 54.13 ± 12.43 mmHg, 54.25 ± 9.44%, 2.33 ± 0.41 L/(min·m^2^), and 11.80 ± 2.97 Wood units, respectively (Table 2).

### 3.3. Comparison of miRNA Expression Levels in the CTEPH and Reference Groups

**Figure 1:**
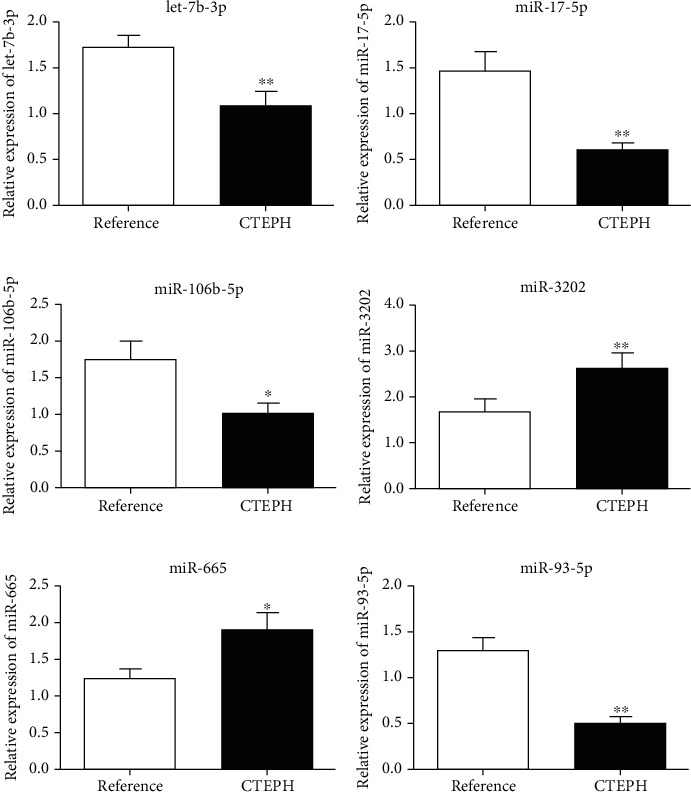
Relative expression of (a) let-7b-3p, (b) miR-17-5p, (c) miR-106b-5p, (d) miR-3202, (e) miR-665, and (f) miR-93-5p in CTEPH patients and the reference group. CTEPH: chronic thromboembolic pulmonary hypertension. ^∗^*P* < 0.05; ^∗∗^*P* < 0.01.

qPCR was adopted to detect the expression levels of let-7b-3p, miR-17-5p, miR-106b-5p, miR-3202, miR-665, and miR-93-5p in the peripheral blood samples from both groups. The peripheral blood expression levels of miR-3202 and miR-665 were significantly higher in patients with CTEPH than in the reference group (^∗^*P* < 0.05; ^∗∗^*P* < 0.01). Conversely, the peripheral blood expression levels of let-7b-3p, miR-17-5p, miR-106b-5p, and miR-93-5p were significantly lower in CTEPH patients than in the reference group (^∗^*P* < 0.05; ^∗∗^*P* < 0.01; Figure 1).

### 3.4. Correlations between miRNA Expression and Changes in Clinical Parameters

A positive correlation was observed between miR-93-5p expression and NT-proBNP level (*r* = 0.831, *P* < 0.05), while a negative correlation was observed between miR-93-5p expression and CI (*r* = −0.861, *P* < 0.05). Additionally, let-7b-3p expression showed a trend toward a positive correlation with mPAP (*r* = 0.811, *P* = 0.096). However, no significant correlations were observed between the expression levels of other miRNAs and clinical parameters (Table 3).

### 3.5. Comparison of miRNA Expression Levels in CTEPH Patients with Different WHO FCs and Risk Stratifications

CTEPH patients were categorized into a WHO FC II group (*n* = 5) and a WHO FC III group (*n* = 3). No significant differences in miRNA expression were observed between the two groups (Table 4). CTEPH patients were further classified into the intermediate-risk or the high-risk groups based on the risk assessment criteria in the 2015 ESC/ERS guidelines [16]. Again, no significant differences in the expression levels of miR-17-5p, miR-665, miR-106-5p, miR-3202, and let-7b-3p were found between the two groups. However, a marginally significant difference in expression was observed for miR-93-5p (0.348 ± 0.156 vs. 0.655 ± 0.133, *P* = 0.060; Table 5).

## 4. Discussion

In the current study, we evaluated the relationships between miRNA expression levels and clinical parameters in patients with CTEPH. The severity of CTEPH was assessed according to miRNA expression as well. Our findings indicate that miR-3202 and miR-665 were upregulated, whereas let-7b-3p, miR-17-5p, miR-106b-5p, and miR-93-5p were downregulated in CTEPH. More importantly, miR-93-5p expression was positively correlated with the serum NT-proBNP concentration and negatively correlated with CI.

CTEPH usually results in progressive exertional dyspnea and right ventricular failure. NT-proBNP is an inactive by-product of proBNP cleavage that occurs under high filling pressures [17]. It remains stable at room temperature and increases to a higher level in pathological conditions [18, 19]. Previous studies suggested that the serum NT-proBNP concentration could reflect the severity of right ventricular function injury, with a higher NT-proBNP level indicating a more severe CTEPH classification [20–22]. In the present study, the associations between miRNA expression levels (let-7b-3p, miR-17-5p, miR-106b-5p, miR-3202, miR-665, and miR-93-5p) and the NT-proBNP concentration was investigated. We observed that the miR-93-5p expression was positively correlated with NT-proBNP concentration, which suggested that miR-93-5p may serve as an indicator of the severity of CTEPH.

Hemodynamic parameters, including mPAP, SvO_2_, CI, and PVR, represent right ventricular function and thus are important indicators reflecting the severity of CTEPH [23]. Among these parameters, CI is an indicator of cardiac pumping function. A decrease in the CI is accompanied by an increase in right ventricular end-diastolic and pulmonary arterial pressure, as well as a decrease in right ventricular work. In severe CTEPH patients, right ventricular dysfunction leads to a CI decrease [24]. In the present study, miR-93-5p expression was negatively correlated with CI, implying that miR-93-5p may be an alternative biomarker for assessing right ventricular function and the severity of CTEPH.

We speculate that miR-93-5p may affect the severity of CTEPH by interacting with NT-proBNP and influencing the CI via myeloid leukemia/hepatitis C pathways in cancer/HTLV-I infection/axon guidance, because pathway enrichment analysis indicated that the top five pathways for the targets of miR-93-5p are chronic myeloid leukemia, hepatitis C, cancer-related pathways, HTLV-I infection, and axon guidance [13]. Previous studies demonstrated that miR-93-5p is involved in multiple pathophysiological processes, including angiogenesis, autophagy, and inflammation. An in vitro study showed that miR-93-5p regulates the function of human endothelial cells through downregulating epithelial protein loss in neoplasm [25]. Vascular endothelial growth factor and the inflammatory cytokine interleukin-8 were reported to be the major targets of miR-93-5p in neuroblastoma cells [26]. In acute myocardial infarction, miR-93-5p was found to have a cardioprotective effect by suppressing Atg7-mediated autophagy and TLR4/NF-*κ*B-mediated inflammatory responses [27]. Increasing evidence indicates that miR-93-5p may play an important role in the regulation of the cardiovascular system. However, the specific mechanism of miR-93-5p in CTEPH needs to be further clarified.

Furthermore, mPAP is an important pulmonary hemodynamic parameter for identifying and managing CTEPH [28, 29]. It can reflect the severity of mosaic perfusion [30]. Guo et al. previously found that let-7b is downregulated in CTEPH patients. miRNA let-7b affects the migration of pulmonary arterial endothelial cells and smooth muscle cells by altering endothelin-1 and transforming growth factor (beta receptor I) expression [31]. In the present study, let-7b-3p expression appeared to be positively correlated with mPAP, although the association did not reach statistical significance. Accordingly, the role of let-7b in the pathogenesis of CTEPH warrants further investigation.

To our knowledge, this is the first study to investigate the relationship between miRNA expression levels and the clinical characteristics of CTEPH. This work expands the current understanding of the roles of miRNAs in the development of CTEPH. miRNAs are ideal candidates for future studies based on their connection with disease severity indicators (e.g., NT-proBNP and CI). Further research including in-depth functional evaluation and protein-level analysis may provide insight into the genetic and molecular mechanisms of CTEPH.

There are several limitations in this study. First, the sample size was relatively small, which may cause deviation in the results. In the current study, we did not detect miRNA expression differences in CTEPH patients with different WHO FC classifications or risk stratifications, possibly due to the limited sample size. Second, miRNA expression was detected in peripheral blood samples. As CTEPH is a multietiological disease, diverse miRNA sources might provide greater insight into the mechanisms of this disease. Third, the underlying mechanisms of the associations between miRNA expression levels and clinical indices need to be further elucidated. A large-scale study is warranted to verify our findings.

## 5. Conclusions

In conclusion, our study suggests that miR-93-5p expression correlates with the severity of CTEPH and could serve as a potential predictor of high-risk CTEPH. The impact of miR-93-5p in the pathogenesis of CTEPH is worth further investigation.

## Figures and Tables

**Table 1:** Demographic and biochemical data of CTEPH patients and the reference group.

Parameters	Reference group (*n* = 8)	CTEPH group (*n* = 8)	*P* value
Gender, female	4	4	-
Age, years	56.13 ± 4.49	61.00 ± 6.82	0.113
Hemoglobin, g/L	137.88 ± 15.52	153.00 ± 18.61	0.099
Hematocrit, %	40.96 ± 3.67	42.84 ± 4.11	0.352
Platelet, 10^6^/L	199.25 ± 40.27	224.00 ± 45.39	0.268
TP, g/L	76.79 ± 4.14	67.15 ± 4.56	0.001
Alb, g/L	46.09 ± 2.79	39.80 ± 2.88	0.001
A : G	1.50 ± 0.18	1.46 ± 0.09	0.603
AST, U/L	27.38 ± 4.90	29.75 ± 18.88	0.736
ALT, U/L	25.12 ± 9.97	30.50 ± 24.78	0.578
LDH, U/L	180.16 ± 27.30	243.75 ± 45.96	0.011
HBDH, U/L	153.00 ± 153.00	214.38 ± 40.20	0.006
TBIL, *μ*mol/L	14.66 ± 4.73	19.88 ± 7.70	0.130
DBIL, *μ*mol/L	4.53 ± 1.46	7.40 ± 3.67	0.071
BUN, mmol/L	5.14 ± 0.73	6.78 ± 2.24	0.082
Cr, *μ*mol/L	69.50 ± 12.81	76.80 ± 13.90	0.295
UA, U/L	299.75 ± 76.62	447.87 ± 111.28	0.008

TP: total protein; Alb: albumin; A : G: albumin : globulin; AST: aspartate transaminase; ALT: alanine transaminase; LDH: lactate dehydrogenase; HBDH: hydroxybutyrate dehydrogenase; TBIL: total bilirubin; DBIL: direct bilirubin; BUN: blood urea nitrogen; Cr: creatinine; UA: uric acid.

**Table 2:** Clinical indicators and hemodynamic data of CTEPH patients.

Parameters	CTEPH group
WHO FC (I-II; III-IV)	5; 3
Disease duration, months	43.88 ± 33.37
6MWD, m	390.06 ± 108.06
NT-proBNP, pg/mL	1142.00 (145.42, 2491.75)
mPAP, mmHg	54.13 ± 12.43
PAWP, mmHg	9.6 ± 2.8
SvO_2_, %	54.25 ± 9.44
CI, L/(min·m^2^)	2.33 ± 0.41
PVR, Wood units	11.80 ± 2.97

WHO FC: World Health Organization function classification; 6MWD: 6-minute walk distance; NT-proBNP: N-terminal pro-B-type natriuretic peptide; mPAP: mean pulmonary arterial pressure; PAWP: pulmonary artery wedge pressure; SvO_2_: mixed venous oxygen saturation; CI: cardiac index; PVR: pulmonary vascular resistance.

**Table 3:** Correlation analysis results for miRNA expression levels and clinical data.

Parameters	let-7b-3p		miR-17-5p		miR-106-5p		miR-3202		miR-665		miR-93-5p	
	*r*	*P* value	*r*	*P* value	*r*	*P* value	*r*	*P* value	*r*	*P* value	*r*	*P* value
NT-proBNP	0.357	0.555	0.612	0.197	0.570	0.237	0.160	0.705	-0.120	0.820	0.831	0.041^**#**^
mPAP	0.811	0.096	0.577	0.230	0.258	0.621	0.295	0.478	-0.076	0.887	0.435	0.389
SvO_2_	-0.597	0.288	-0.669	0.146	0.647	0.165	-0.355	0.388	0.276	0.596	-0.725	0.103
CI	-0.279	0.649	0.111	0.833	0.138	0.794	-0.150	0.723	-0.083	0.876	-0.861	0.027^∗^
PVR	0.675	0.211	0.524	0.286	0.255	0.625	0.070	0.869	-0.402	0.429	0.682	0.135

NT-proBNP: N-terminal pro-B-type natriuretic peptide; mPAP: mean pulmonary arterial pressure; SvO_2_: mixed venous oxygen saturation; CI: cardiac index; PVR: pulmonary vascular resistance. ^#^miR-93-5p and NT-proBNP are significantly positively correlated. ^∗^miR-93-5p and CI are significantly negatively correlated.

**Table 4:** Comparison of miRNA expression between CTEPH patients with different WHO FCs.

miRNA	WHO FC II group (*n* = 5)	WHO FC III group (*n* = 3)	*P* value
miR-17-5p	0.360 ± 0.064	1.066 ± 0.379	0.227
miR-665	2.357 ± 0.906	1.024 ± 0.023	0.122
miR-93-5p	0.437 ± 0.219	0.630 ± 0.178	0.346
miR-106-5p	0.500 ± 0.480	1.519 ± 0.591	0.080
miR-3202	2.609 ± 1.684	2.556 ± 0.447	0.950
let-7b-3p	0.964 ± 0.694	1.296 ± 0.663	0.632

**Table 5:** Comparison of miRNA expression between CTEPH patients with different risk stratifications.

miRNA	Intermediate-risk CTEPH (*n* = 4)	High-risk CTEPH (*n* = 4)	*P* value
miR-17-5p	0.357 ± 0.078	0.834 ± 0.483	0.167
miR-665	1.969 ± 0.576	1.856 ± 1.440	0.905
miR-93-5p	0.348 ± 0.156	0.655 ± 0.133	0.060
miR-106-5p	0.545 ± 0.667	1.239 ± 0.740	0.329
miR-3202	2.282 ± 1.752	2.897 ± 0.773	0.544
let-7b-3p	0.964 ± 0.694	1.296 ± 0.663	0.632

## Data Availability

The datasets generated and analyzed in the present study are available from the corresponding author upon reasonable request.

## Supplementary Materials

Supplementary materials Table S1: primer sequences used for qPCR detection of miR-let-7b-3p, miR-17-5p, miR-106b-5p, miR-3202, miR-665, and miR-93-5p expression.
