# Supplementary material for: Expression of miR-93-5p as a Potential Predictor of the Severity of Chronic Thromboembolic Pulmonary Hypertension
Source: Biomed Res Int. 2021 Apr 17;2021:6634417. doi: 10.1155/2021/6634417 (PMC8075669; doi:10.1155/2021/6634417)
Supplement: Supplementary materials — Table S1: primer sequences used for qPCR detection of miR-let-7b-3p, miR-17-5p, miR-106b-5p, miR-3202, miR-665, and miR-93-5p expression. [file 6634417.f1.pdf]

Table S1. Primer sequences used for qPCR detection of miR-let-7b-3p, miR-17-5p, miR-106b-5p, miR-3202, miR-665, and miR-93-5p expression

Primer	Sequence (5'-3')
hsa-let-7b-3p-RT	GTCGTATCCAGTGCAGGGTCCGAGGTA TTCGCACTGGATACGACGGGAAG
JH-hsa-let-7b-3p-F	GCGCCTATACAACCTACTGC
hsa-miR-17-5p-RT	GTCGTATCCAGTGCAGGGTCCGAGGTAT TCGCACTGGATACGACCTACCT
JH-hsa-miR-17-5p-F	GCCAAAGTGCTTACAGTGC
hsa-miR-106b-5p-RT	GTCGTATCCAGTGCAGGGTCCGAGGTAT TCGCACTGGATACGACATCTGC
JH-hsa-miR-106b-5p-F	GCGCTAAAGTGCTGACAGT
hsa-miR-3202-RT	GTCGTATCCAGTGCAGGGTCCGAGGTAT TCGCACTGGATACGACATTAAA
JH-hsa-miR-3202-F	GCTGGAAGGGAGAAGAGC
hsa-miR-665-RT	GTCGTATCCAGTGCAGGGTCCGAGGTAT TCGCACTGGATACGACAGGGGC
JH-hsa-miR-665-F	GCGCACCAGGAGGCTGAG
hsa-miR-93-5p-RT	GTCGTATCCAGTGCAGGGTCCGAGGTAT TCGCACTGGATACGACCTACCT
JH-hsa-miR-93-5p-F	GCCAAAGTGCTGTTCGTGC
U6-hF	CTCGCTTCGGCAGCACA
U6-hR	AACGCTTCACGAATTTGCGT
Universal downstream primer	GTGCAGGGTCCGAGGT
